# Clinical Utility of Ultrasound Imaging for Measuring Anterior Thigh Thickness after Anterior Cruciate Ligament Injury in an Individual Patient to Assess Postsurgery Outcome

**DOI:** 10.1155/2023/6672951

**Published:** 2023-10-23

**Authors:** Filippo Mechelli, Richard Bayford, Hemda Garelick, Maria Stokes, Sandra Agyapong-Badu

**Affiliations:** ^1^Private practice, Urbino, Italy; ^2^Faculty of Science and Technology, Department of Natural Sciences, Middlesex University, London, UK; ^3^School of Health Sciences, University of Southampton, Southampton, UK; ^4^Centre for Sport, Exercise and Osteoarthritis versus Arthritis, Southampton, UK; ^5^Southampton NIHR Biomedical Research Centre, UK; ^6^School of Sport, Exercise and Rehabilitation Sciences, University of Birmingham, Edgbaston, Birmingham, UK

## Abstract

The present study investigated the clinical utility of ultrasound imaging (USI) for assessing changes in an individual's quadriceps muscle and subcutaneous fat (SF) thickness of the anterior thigh and their relative proportions. A patient was studied prior to and after anterior cruciate ligament reconstruction (ACLR) surgery and during rehabilitation. This case study involved an 18-year-old female recreational athlete with a complete tear of the anterior cruciate ligament (ACL). Tissue thickness (SF and quadriceps muscle) was measured from transverse USI of the anterior thigh before surgery, at weekly intervals during 12 weeks of postsurgery, and then every 2 weeks for the following 12 weeks (total of 21 measurement sets). Statistically significant differences presurgery to postrehabilitation were found for muscle thickness (*p* = 0.04) and SF tissue thickness (*p* = 0.04) measurements. There was no difference in muscle to fat ratio (*p* = 0.08). Changes in measurements greater than the reported minimal detectable change (MDC) demonstrate the sensitivity of the USI technique as an objective tool to assess clinically useful changes in an individual's anterior thigh muscle thickness post-ACLR surgery and during rehabilitation.

## 1. Introduction

Rupture of the anterior cruciate ligament (ACL) is among the most common and economically costly sport injuries [1]. Injuries to the ACL frequently require surgery and extensive rehabilitation resulting in an economic burden on society caused by absence from work, reduced productivity, and associated health care costs [2, 3]. Surgical management is currently the preferred treatment for ACL injuries in the UK, with a conservative estimated £63 million (*n* = 15,000) in costs for ACL reconstruction to the NHS in 2015 [4]. Prehabilitation before considering surgery, particularly with isolated ACL tears without comorbidity, is reported to reduce ACL surgery by up to 50% [5].

Quadriceps muscle atrophy and weakness are usually reported in patients after knee surgery and may persist postoperatively for long periods [6, 7], causing a reduction of physical function [8–12], a possible dysfunction of movement patterns [13–18], and an increased risk of reinjury [19–21]. There are inconsistencies in guidelines for ACL reconstruction (ACLR) rehabilitation [22, 23], and 80% of hospital orthopaedic departments within London, UK, have their own ACL rehabilitation guidelines [24], resulting in significant variations and little data on the effect of rehabilitation regimens. ACLR rehabilitation progression should be tailored according to objective data and measurements and not merely on time or protocols.

Physical therapy plays a key role in the rehabilitation process to achieve beneficial clinical outcomes [25]. The clinical assessment of quadriceps femoris muscle bulk has been traditionally performed visually, by observing the contours of the thigh and by measuring limb girth with a tape measure [26, 27]. Visual observation and comparison of the thighs are known to underestimate loss of quadriceps' cross-sectional area (CSA) by 22-33% on the injured side [26]. Specifically, measuring limb girth with an anthropometric tape measure to estimate quadriceps' size involves considering all muscles of the thigh, as well as bone, subcutaneous fat (SF), and all the other anatomical structures not related to the anterior thigh compartment. This approach could lead to errors in estimating muscle size [26–28], in addition to effects of inter- and intraoperator reliability [29]. An accurate and objective assessment of quadriceps muscle atrophy is a powerful tool to guide physiotherapy care [30].

Musculoskeletal ultrasound imaging (USI) is used to assess the morphology, CSA, and thickness of muscles and other neuromusculoskeletal structures [29, 31]. The technique provides a rapid, accurate, safe, portable, noninvasive method of obtaining objective measurements, and it is much less expensive than computed tomography (CT) and magnetic resonance imaging (MRI).

Reliability of measurements between investigators [32] and test retest reliability [32] and criterion validity against the gold standard of MRI [33] were recently reported for USI in measuring quadriceps muscle and SF thickness of the anterior thigh. The sensitivity of the USI measurements over a 2-year period has also been reported [34], advancing the application of the USI technique to longitudinal studies.

As highlighted earlier, there is little consensus on the management of ACL injuries, and the present study is aimed at demonstrating how USI can provide objective evidence regarding the clinical outcomes following ACLR surgery and inform care for acute ACL patients. Specifically, the study implemented and assessed the clinical utility of USI for measuring anterior thigh tissue thickness after ACL injury, ACLR intervention, and during rehabilitation, to indicate how USI can be used to monitor an individual patient (*n* of 1) objectively [35].

## 2. Materials and Methods

### 2.1. Participant

An 18-year-old female recreational athlete who suffered a complete tear of the left ACL and underwent ACLR surgery was studied. The protocol was approved by Middlesex University Research Ethics Committee (#14872/2020). The study was conducted in accordance with the ethical standards of the Declaration of Helsinki, as revised in 2013 [36]. Written informed consent was obtained from the participant after full explanation of the aims and procedures and after providing her with a written information sheet. The participant's rights were protected at all times during this study.

### 2.2. Procedure

Transverse B-mode images of the anterior thigh were acquired using an ultrasound scanner (MyLab25 Gold; Esaote, Genova, Italia) with a 7.5 MHz linear transducer (40 mm length). Ultrasound images were obtained with the participant lying in a supine position with the hips in neutral and knees fully extended, with the support of sandbags at the ankles to avoid rotation. Measurements were performed at a site two-thirds of the distance between the anterosuperior iliac spine and the superior pole of the patella in the sagittal plane [32], and the site was marked on the skin with a nontoxic pen. Images were acquired coating the transducer with a generous amount of ultrasound transmission gel and applying minimal pressure to the contact point with the skin to avoid compression of the underlying tissues [34].

Thigh circumference measurements were taken at the same site using a tape measure.

Ultrasound images and thigh circumference measurements were acquired at 4, 5, and 6 weeks after injury presurgery (6 weeks after the injury and 1 week before surgery were the same day), at weekly intervals post-ACLR surgery to 12 weeks, and then every 2 weeks for the following 12 weeks. In all, a total of 21 measurement sets were taken.

### 2.3. Ultrasound Imaging Data Processing

**Figure 1:**
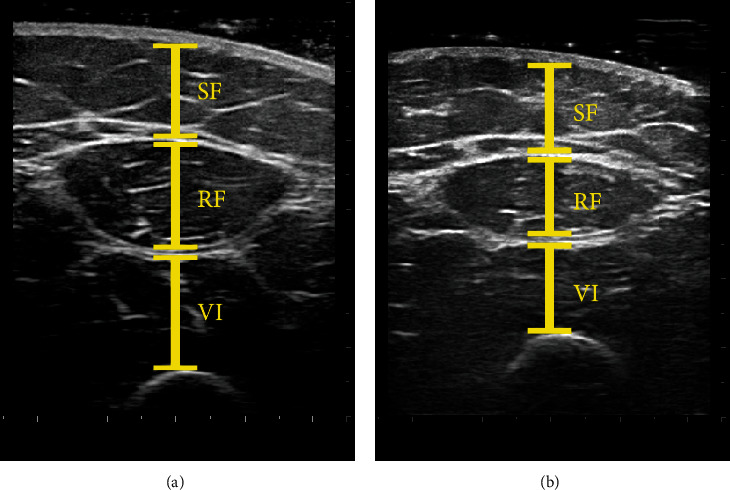
Ultrasound images of the anterior thigh 3 weeks post-ACLR of the (a) uninjured limb and (b) injured limb. SF = subcutaneous fat; RF = rectus femoris muscle; VI = vastus intermedius muscle.

Ultrasound images were analysed offline using ImageJ software (available from https://imagej.nih.gov/ij/). SF thickness was included between the skin and the outside edge of the superficial fascial layer of rectus femoris (RF); thickness of RF and vastus intermedius (VI) were determined between the inside edges of each muscle border, excluding perimuscular fascia (Figure 1).

### 2.4. Study Design

This was a longitudinal *n*-of-1 study [35] over a period of 27 weeks.

### 2.5. Data Analysis

Data were analysed using SPSS 25 (SPSS Inc., Chicago, IL). Student's *t*-test was used to compare mean differences in the thickness of the anterior thigh tissues between the 3 measurements acquired before ACLR surgery and the last 3 measurements of the final period of rehabilitation.

Holm's correction was used to adjust the *p* values for multiple testing within an individual positive rate to 5% [37]. Differences were considered statistically significant for *p* values less than 0.05.

Visual observation analysis of data presented graphically was performed to evaluate changes in trend across the different phases.

Changes in measurement values between different phases were compared to minimal detectable change (MDC) values reported in literature [32], to assess if differences were greater than the error associated with the measurement technique. The MDC values were generated in a previous study [32] by the lead investigator (FM), who acquired all the scans and established between-day reliability [32], with intraclass correlation coefficient (ICC3.2) values of 0.96 for muscle and 0.98 for SF. MDC values of the same reliability study were 3.6 mm for total muscle thickness (RF+VI) and 1.3 mm for SF [32]. Retrospective analysis of the data of the same test-retest reliability study [32] produced intraclass correlation coefficient (ICC3.2) values of 0.93 for RF and 0.89 for VI and MDC values of 2.5 mm for RF and 3.3 mm for VI.

Percentage difference between uninjured and injured side was calculated as [(uninjured limb − injured limb)/uninjured limb] × 100.

## 3. Results

Changes in anterior thigh tissue thickness between pre-ACLR surgery and 24 weeks post-ACLR were evident on all data presentation and analysis methods used, which concurred with one another.

### 3.1. Statistical Comparisons over Time

Total muscle thickness (RF+VI) decreased from pre-ACLR to 3 weeks post-ACLR and then began increasing at 6 weeks post-ACLR, with a constant increment to the end of the study period (Table 1). SF increased gradually postsurgery and recorded a dip towards the end of the study period.

From pre-ACLR until 3 weeks post-ACLR, VI muscle thickness decreased and then remained almost stable until 12 weeks post-ACLR. VI thickness then increased during the last 12 weeks of the study period exceeding the preintervention value at the final measurement. For RF muscle thickness, a decrease from preintervention to 1 week postintervention was observed, which then slowly began to increase and remained stable between 6 and 12 weeks postintervention, with a further increase in size during the last 12 weeks of the rehabilitation period that exceeded the preoperative measurement.

A separate evaluation of the two muscles (RF and VI), rather than the sum of both, enables assessment of selective atrophy of one of the two muscles. There was a greater decrease in VI pre- to post-ACLR in comparison to RF. Both RF and VI thicknesses exceeded the presurgery values by the end of the study period. A more pronounced and persistent atrophy was evident in VI rather than in RF, and a faster recovery of RF was observed.

Thigh girth measurements in the present study (Table 1) remained fairly consistent throughout the study.

Statistical differences in anterior thigh tissue thickness measurements between pre-ACLR surgery and postrehabilitation are shown in Table 2. There were statistically significant differences between muscle thickness (*p* = 0.04) and SF tissue thickness (*p* = 0.04) and thigh girth (*p* = 0.03) measurements taken prior to ACLR intervention and postrehabilitation, while there was no statistically significant difference in muscle to fat ratio (*p* = 0.08).

### 3.2. Trends in Outcome Measures

**Figure 2:**
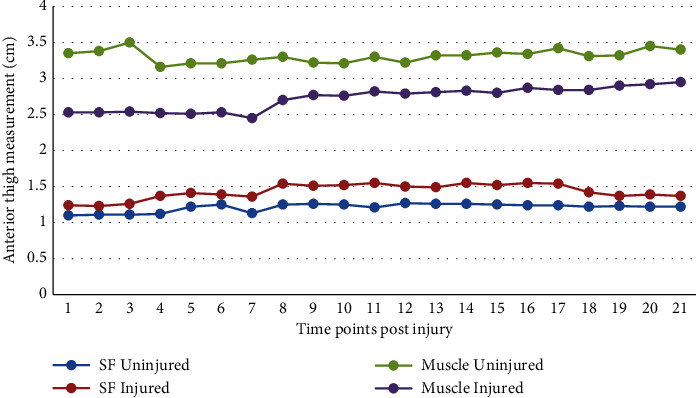
Trend in anterior thigh muscle and subcutaneous tissue thickness measurements following anterior cruciate ligament injury across the study period showing the healthy control level and injured limb. Muscle = vastus intermedius+rectus femoris; SF = subcutaneous fat.

The trend in anterior thigh muscle and SF tissue thickness of the injured and uninjured limb measurements across the whole study period can be observed in Figure 2. At the baseline (time point 1), there was evident atrophy of quadriceps in the injured limb compared with the uninjured one. A decrease in muscle thickness at 1 week post-ACLR (time point 4) is more evident for the uninvolved limb (healthy control) than the injured limb, the latter being already atrophied due to the ACL injury itself. Muscle thickness of the injured limb began to increase after 4 weeks post-ACLR (time point 7), while muscle thickness in the uninjured began to increase from 2 weeks post-ACLR (time point 5). Muscle thickness of the injured limb exceeded the preoperative value at 24 weeks post-ACLR (time point 21), and that of the uninjured limb returned to the preoperative value.

SF tissue thickness of the anterior thigh of the injured limb was greater compared with the contralateral uninjured limb throughout the study period, and both remained stable (Figure 2).

### 3.3. Comparison with MDC

The changes in anterior thigh measurements of the injured and uninjured limb, compared with MDC values between preoperative to postoperative, preoperative to postrehabilitation (24 weeks post-ACLR), and postoperative to postrehabilitation, are shown in Table 3.

Measurements greater than the MDC were found in the injured limb pre-ACLR surgery to postrehabilitation, for total muscle thickness (RF+VI) and for RF, while post-ACLR surgery to postrehabilitation, measurements greater than the MDC value were found for total muscle thickness (RF+VI) and for both VI and RF measured separately (Table 3).

Differences in measurements in the injured limb between presurgery and postsurgery for muscle tissue thickness (total and separately for RF and VI), and differences in SF tissue thickness measurements in all the periods assessed, were less than MDC values and so inside the error associated with the measurement technique (Table 3).

For the uninjured limb, measurements greater than the MDC were only found for total muscle thickness (RF+VI) pre-ACLR surgery to post-ACLR surgery and post-ACLR surgery to postrehabilitation.

Differences in anterior thigh tissue thickness that were greater than MDC values between injured and uninjured lower limbs were found for muscle at presurgery and postsurgery but not postrehabilitation (Table 4).

Differences greater than MDC values in SF tissue thickness between injured and uninjured limbs were found presurgery and postsurgery (Table 4). Percentage between-side difference in muscle thickness was greatest preoperatively (-32%) and reduced postoperatively until reducing below 10% at the end of rehabilitation, when the difference was also below the MDC. Large percentage (>10%) between-side differences in SF tissue thickness were found, with the involved limb being greater, returning to near 10% postrehabilitation but still above the MDC.

## 4. Discussion

The present findings confirm the presence of quadriceps muscle atrophy that is known to occur rapidly after ACLR surgery [6, 38, 39] and how USI can be used to monitor recovery. A separate assessment of RF and VI (Table 1) revealed selective atrophy, where reduced thickness was more marked in VI than RF postoperatively. The use of the existing MDC values of tissue thickness enabled objective assessment of abnormality and recovery using USI in the individual patient.

The finding of selective atrophy of VI confirmed a recent study [38] where quadriceps muscle thickness was measured using USI in 14 patients aged 30.4 ± 5.9 years. Measurements were taken 1 hour prior and 48-72 hours after ACLR, which showed a significant decrease in VI thickness compared with presurgery values and compared with the other heads of quadriceps femoris muscle. The underlying mechanism of selective atrophy of VI after ACLR surgery is unknown; therefore, further studies are needed to investigate and clarify the possible causes to minimise VI atrophy using targeted rehabilitation approaches.

To understand the slower recovery of VI compared to RF after ACLR surgery, the anatomy and function of the quadriceps muscle can be considered [40]. It can be observed that VI is a monoarticular muscle and acts just at knee level, while RF is a biarticular muscle and acts both as a knee extensor and hip flexor. It may be possible that the dual joint actions of RF cause it to be stimulated consistently during rehabilitation even when the knee is kept at full extension, thus resulting in earlier recovery of muscle thickness than VI. The surgical access through the knee joint capsule could play a role inducing an inhibition of the articularis genus muscle that inserts into the synovial membrane of the joint capsule and the suprapatellar bursa, and occasionally, its distal muscle fibres are blended with the suprajacent fibres of VI [41, 42]. The articularis genus muscle shares the same blood supply with VI via the deep circumflex branch of the femoral artery and the same innervation via the deep intermuscular branches of the femoral nerve [41, 42]. The close anatomical links between articularis genus and VI could explain the interaction, with the underlying mechanism between the two muscles being more complicated than has been previously assumed [41, 42]. Further studies are required to better understand the role of this mechanism of interaction and to investigate its possible implication during surgical knee procedures.

The present study also found a diminished quadriceps thickness on the contralateral uninjured side from the pre- to postsurgery period, which may be attributed to reduced mobility in the perioperative period. Another possible cause of a transient bilateral lower limb weakness is spinal anaesthesia, due to motor and sensory inhibition, anaesthetic neurotoxicity, and neuroendocrine stress response [43]. The mechanism by which anaesthesia could induce muscle weakness/atrophy, by influencing the neuroendocrine stress response, is unclear [43]. However, reduced mobility is the more likely explanation for bilateral atrophy.

Thigh girth measurements (Table 1) remained consistent throughout the study period, despite changes in muscle and SF thickness measurements, demonstrating limitations in the sensitivity of thigh girth as an outcome measure to monitor SF and/or muscle changes, confirming previous studies [26–28]. SF tissue thickness (Table 4) was greater on the injured side contributing to thigh circumference, also demonstrating inaccuracies in thigh girth for estimating differences in muscle size. It is logical that by measuring thigh girth using a tape measure, the measurement includes all anatomical structures of the thigh and not only the specific structures of interest. At best, the measure provides an estimate of the global state of the entire thigh compared to the uninjured limb but is far from being an accurate measurement.

The MDC values used to compare the data from the present case study participant were derived from a test-retest reliability study [32], which involved 24 participants (12 females, 12 males) aged 48.91 ± 9.78 (36-64) years. Changes in values smaller than the MDC, 3.6 mm for total muscle thickness (RF+VI) and 1.3 mm for SF thickness [32], are likely caused by random measurement error. The retrospective analysis of data from the same between-day reliability study [32] to obtain MDC values for RF (2.5 mm) and VI (3.3 mm) allowed specific evaluation of each muscle separately, highlighting a selective atrophy, which was intended to enable specific and customized physiotherapy care. A selective change in RF thickness of the injured lower limb between the pre-ACLR surgery and postrehabilitation periods was observed (Table 3), with changes in the other periods being either below or above the MDC in both VI and RF muscles.

The present 18-year-old participant was younger than the group from which the MDC values were generated.

The age of the participant, the quality of the ultrasound image that is associated with the echogenicity of the individual's tissues, and the error associated with the measurement technique itself represent important variables in determining MDC values. In a recent study [44] of 12 young male adults aged 26.5 ± 3.9 years, the MDC value of RF thickness (VI and SF were not measured), for the test-retest reliability, was 2.0 mm. This MDC value from younger people may be more appropriate, but the MDC was not for total muscle thickness, and the number of participants studied [44] was smaller (*n* = 12) compared to the study (*n* = 24) used for MDC values in the present study [32]. Further studies could investigate the MDC values using USI in measuring anterior thigh tissue thickness in a younger age group and also differentiate the values for both RF and VI.

A recent study using USI in 26 patients who underwent ACLR surgery revealed a reduction in RF CSA from presurgery to 9 weeks postsurgery (*p* < 0.01), followed by an increase of CSA from 9 weeks to 9 months postsurgery (*p* = 0.03) [39].

Reduction in RF CSA was also recorded in the uninjured limb from surgery to 9 weeks postsurgery (*p* < 0.01), with a complete return to the preoperative CSA at 9 months postsurgery, when the injured limb failed to recover [39]. Differences between the cited study [39] and the present study are that we measured VI as well as the RF muscle (showing selective changes) and SF tissue of anterior thigh and measured muscle thickness, which is easier and faster than measuring CSA, although less reflective of muscle mass.

Limitations of the present study are mainly intrinsic to the type of study design (*n* of 1) [35], concerning external validity and replicability and providing low level of evidence. The investigator conducting the USI underwent training and established their reliability [32]. External validity and generalisability were not addressed, but these were not part of the aims of the present study. Rather, the aim was to provide clinically useful measurements to enable personalized patient care that could be delivered with precision. Another limitation was that muscle strength was not measured directly and USI only provides an indication of force. It is generally accepted that the relationship between muscle size and strength is positive, but the level of correlation varies between muscles and also in response to strength training [45]. This dissociation between the two variables with training involves neural motor control and/or cellular and molecular adaptations of muscle fibres [46]. Such neural adaptation could possibly explain the lack of increase in muscle thickness found between 6 and 12 weeks of rehabilitation (Table 1), at a time when strength would be expected to increase. Another possibility is that the rehabilitation programme may not have provided sufficient stimulus to induce continued increase muscle size or, indeed, strength, which would need to be measured to determine this. However, strength testing does not allow selective changes between muscles to be recognised, as demonstrated by the present findings using USI.

Potential clinical implications of the present study are that the USI technique could be used to assess clinically useful changes of RF and VI muscle thickness in an individual patient post-ACLR surgery, enabling individualized and tailored optimal clinical care.

Skeletal muscle wasting and atrophy are commonly reported in critically ill patients and occur rapidly during the first week of critical illness, having significant implications on patient outcomes [47–50]. Critical illness patients suffer severe muscle atrophy and impaired muscle function, with increased morbidity and health care costs and poorer quality of life [51, 52]. Monitoring skeletal muscle size using USI in critically ill patients at the bedside is increasingly used, as it has proved to be an accurate and reliable tool to assess muscle changes [47–54].

The RF muscle is typically monitored, but the present observations suggest that VI muscle could be more sensitive to atrophy than RF (Table 1), so it may be preferable to include VI in the assessment. However, the disuse in intensive care patients without lower limb injuries may result in atrophy through a different mechanism to that seen with ACL injuries, which may involve inhibitory reflex responses from articular/periarticular receptors [55].

Further studies are needed to investigate a greater susceptibility to atrophy of VI compared to RF with different causal mechanisms and the potential use of USI as an indicator of the early muscle atrophy process.

## 5. Conclusions

The present findings demonstrate that it is possible to measure statistically significant differences in USI measurements of anterior thigh muscle and SF tissue thickness in an individual over time, using comparison with MDC values. Measurements taken prior to ACLR surgical intervention and postrehabilitation showed greater reductions in VI than RF muscle thickness, indicating selective atrophy. These findings confirm the utility of the USI technique as an accurate tool with good sensitivity for monitoring effects of surgery and physiotherapy rehabilitation in an individual patient.

## Figures and Tables

**Table 1:** Anterior thigh tissue thickness measurements on the injured and uninjured limbs following ACLR.

18-year-old female: height, 1.7 m; weight, 58 kg; BMI, 20.1 kg/m^2^; dominant side, right lower limb (uninjured)										
	Uninjured lower limb (cm)					Injured lower limb (cm)				
	SF	RF	VI	M/F	TG	SF	RF	VI	M/F	TG
Pre-ACLR (T3)	1.11	1.71	1.79	3.15	47	1.26	1.11	1.28	1.89	45
1 wk post-ACLR (T4)	1.12	1.56	1.60	2.82	47	1.37	1.11	1.15	1.65	45
3 wks (T6)	1.25	1.55	1.66	2.57	47	1.39	1.19	1.14	1.68	45
6 wks (T9)	1.26	1.52	1.70	2.56	47.5	1.51	1.47	1.26	1.81	46
12 wks (T15)	1.25	1.57	1.79	2.69	47.5	1.52	1.50	1.28	1.83	46.4
24 wks (T21)	1.22	1.61	1.79	2.79	47.5	1.37	1.57	1.58	2.29	47.2

BMI = body mass index; wks = weeks; T = time points of interest; SF = subcutaneous fat tissue; RF = rectus femoris; VI = vastus intermedius; M/F = muscle to fat ratio calculated as [(rectus femoris + vastus intermedius)/subcutaneous fat] no units; TG = thigh girth.

**Table 2:** Examination of differences in anterior thigh tissue thickness between pre-ACLR surgery and postrehabilitation on the injured limb.

Tissue thickness (cm)	Presurgery	Postrehab	Paired mean diff	SD	SEM	Lower	Upper	*t*	df	Holm's adjusted *p* value
SF thickness (cm)	1.24 ± 0.02	1.38 ± 0.01	-0.14	0.03	0.01	-0.19	-0.07	-9.18	2	0.04^∗^
Muscle thickness (cm)	2.48 ± 0.08	3.09 ± 0.05	-0.61	0.13	0.07	-0.93	-0.29	-8.34	2	0.04^∗^
Muscle to fat ratio	1.99 ± 0.08	2.24 ± 0.05	-0.25	0.13	0.08	-0.58	0.07	-3.33	2	0.08
Thigh girth (cm)	45.17 ± 0.29	47.2 ± 0.01	-2.03	0.29	0.17	-2.75	-1.32	-12.2	2	0.03^∗^

SD = standard deviation; SEM = standard error of the mean; df = degrees of freedom. ^∗^Significant 2-tailed; *p* < 0.05. Presurgery = last 3 measurements prior to ACLR. Postrehab = last 3 measurements at the final period of rehabilitation.

**Table 3:** Anterior thigh tissue thickness measurements of the injured and uninjured lower limbs compared to MDC values obtained from a reliability study^32^.

			Difference compared to MDC (cm) VI = 0.33; RF = 0.25; MT = 0.36; SF = 0.13
Injured limb	Presurgery (cm)	Postsurgery (cm)	
VI	1.28	1.15	0.13 < MDC
RF	1.11	1.11	0 < MDC
MT	2.39	2.26	0.13 < MDC
SF	1.26	1.37	0.11 < MDC
	Presurgery (cm)	Postrehabilitation (cm)	
VI	1.28	1.58	0.3 < MDC
RF	1.11	1.57	0.46 > **MDC**^∗^
MT	2.39	3.15	0.76 > **MDC**^∗^
SF	1.26	1.37	0.11 < MDC
	Postsurgery (cm)	Postrehabilitation (cm)	
VI	1.15	1.58	0.43 > **MDC**^∗^
RF	1.11	1.57	0.46 > **MDC**^∗^
MT	2.26	3.15	0.89 > **MDC**^∗^
SF	1.37	1.37	0 < MDC
Uninjured limb	Presurgery (cm)	Postsurgery (cm)	
VI	1.79	1.60	0.19 < MDC
RF	1.71	1.46	0.25 < MDC
MT	3.50	3.06	0.44 > **MDC**^∗^
SF	1.11	1.12	0.01 < MDC
	Presurgery (cm)	Postrehabilitation (cm)	
VI	1.79	1.79	0 < MDC
RF	1.71	1.61	0.10 < MDC
MT	3.50	3.40	0.10 < MDC
SF	1.11	1.22	0.11 < MDC
	Postsurgery (cm)	Postrehabilitation (cm)	
VI	1.60	1.79	0.19 < MDC
RF	1.46	1.61	0.15 < MDC
MT	3.06	3.40	0.44 > **MDC**^∗^
SF	1.12	1.22	0.10 < MDC

VI = vastus intermedius; RF = rectus femoris; MT = muscle thickness VI + RF; SF = subcutaneous fat; MDC = minimal detectable change; ^∗^ and bold = values greater than MDC.

**Table 4:** Differences in anterior thigh tissue thickness between injured and uninjured lower limbs compared to MDC values.

Anterior thigh thickness (cm)	Injured (cm)	Uninjured (cm)	Difference compared to MDC MT = 0.36; SF = 0.13	Percentage between-side difference
MT				
T3	2.39	3.50	1.11 > **MDC**^∗^	-32%
T4	2.26	3.16	0.90 > **MDC**^∗^	-28%
T21	3.15	3.40	0.25 < MDC	-7%
SF				
T3	1.26	1.11	0.15 > **MDC**^∗^	+14%
T4	1.37	1.12	0.25 > **MDC**^∗^	+22%
T21	1.37	1.22	0.15 > **MDC**^∗^	+12

MT = muscle thickness: vastus intermedius+rectus femoris; SF = subcutaneous fat; MDC = minimal detectable change; ^∗^ and bold = values greater than MDC; T3 = 1 week presurgery; T4 = 1 week postsurgery; T21 = 24 weeks postsurgery.

## Data Availability

Data will be made available on request.
